# The impact of mindfulness intervention on emotion regulation in athletes: a meta-analysis

**DOI:** 10.3389/fpsyg.2026.1875000

**Published:** 2026-07-23

**Authors:** Jiaxuan Li, Jiaxi Lu, Dongmei Fang

**Affiliations:** 1 School of Physical Education, Jiangsu Normal University, Xuzhou, China; 2 Sports Operation and Management, Guangxi College of Sports Education, Nanning, China

**Keywords:** athlete, emotion regulation, meta-analysis, mindfulness, sport

## Abstract

**Introduction:**

Athletes frequently encounter considerable emotional challenges during high-intensity training and competition. Effective emotion regulation is essential not only for athletic performance but also for long-term mental health and career longevity. Although mindfulness interventions have been increasingly applied in sport psychology, their effects on athletes’ emotion regulation have not been comprehensively evaluated. This meta-analysis therefore examined the effects of mindfulness interventions on emotion regulation among athletes.

**Methods:**

Seven eligible studies involving 273 participants were synthesized to evaluate the effects of mindfulness interventions on athletes’ emotion regulation.

**Results:**

Mindfulness interventions produced a statistically significant, moderate effect on athletes’ emotion regulation (Hedges’ *g* = 0.81, 95% CI [0.51, 1.11], *p* < 0.0001). Egger’s regression test was not statistically significant (*p* > 0.05); however, this finding provides limited reassurance because the test has low statistical power when fewer than 10 studies are included.

**Discussion:**

The overall estimate was based on only seven studies, and the direct comparison between Mindfulness-Acceptance-Commitment training and Psychological Skills Training was based on only three studies. The findings should therefore be considered preliminary and require confirmation in larger, higher-quality randomized controlled trials. Future research should examine diverse athlete populations and intervention conditions to clarify how mindfulness-based and traditional psychological training approaches contribute to athletes’ emotion regulation and psychological functioning.

**Systematic review registration:**

PROSPERO. Registration number: CRD420261340782. https://www.crd.york.ac.uk/PROSPERO/view/CRD42026134078

## Introduction

In order to help athletes achieve optimal competitive and psychological states during competition, sport psychologists have conducted extensive research on effective intervention methods and target populations (Alecu and Onea, 2025). Emotion regulation plays a central role in athletes’ psychological functioning and competitive performance (Regborn et al., 2025). Emotion regulation refers to the process by which individuals influence what emotions they experience, when they experience them, and how these emotions are felt and expressed (McRae and Gross, 2020). It encompasses both explicit regulation and implicit regulation. For athletes, emotions exert a significant influence on their sport performance. Athletes frequently encounter high-pressure situations, including training demands, competitive uncertainty, and external expectations, which can evoke intense emotional responses such as anxiety, frustration, and fear of failure. The ability to regulate these emotions effectively shapes not only immediate performance outcomes but also long-term mental health and career sustainability (Gross, 2015). Adaptive strategies, such as cognitive reappraisal and acceptance, support athletes in maintaining focus, recovering quickly from mistakes, and sustaining motivation under adversity. By contrast, reliance on maladaptive strategies, such as suppression or rumination, has been linked to heightened stress, impaired decision-making, and increased risk of burnout. With growing recognition of the value of mental skills training, enhancing athletes’ capacity to regulate emotions has become a key focus of contemporary sport psychology research and practice (Tingaz and Altun Ekiz, 2025).

From a theoretical perspective, Cognitive Appraisal Theory of Emotion and the Transactional Model of Stress and Coping provide a conceptual framework for understanding how athletes’ interpretations of competitive demands influence their emotional responses and performance outcomes (Meijen et al., 2020). According to these models, athletes cognitively appraise competitive situations as either challenges or threats. Threat appraisals often elicit maladaptive emotions such as anxiety and fear, whereas challenge appraisals are associated with optimal arousal and superior performance under pressure.

Mindfulness may play a critical role in this appraisal process by cultivating nonjudgmental awareness of internal experiences and fostering acceptance of competition-related stressors (Kabat-Zinn, 1994). Through this mechanism, mindfulness enables athletes to reframe stressful events as manageable, thereby reducing competitive anxiety and facilitating adaptive emotion regulation that supports optimal performance. Mindfulness-based interventions (MBIs) are believed to enhance emotion regulation through multiple mechanisms. First, mindfulness practice strengthens attentional control and present-moment awareness, allowing individuals to more readily perceive their thoughts and emotions and to identify distressing feelings that require regulation (Josefsson et al., 2017). Second, common mindfulness exercises such as observing and describing emotions promote greater emotional awareness and differentiation, which helps individuals recognize and distinguish among various emotional experiences and reduce automatic affective reactions and intensity (Raugh and Strauss, 2024). Third, the nonjudgmental and accepting attitude cultivated through mindfulness fosters greater tolerance and willingness to experience negative emotions without suppressing or avoiding them (Gratz and Tull, 2010), thereby enhancing resilience to fear of failure and other aversive emotional states.

Prominent mindfulness-based programs in sport include the Mindfulness–Acceptance–Commitment (MAC) approach and the Mindful Sport Performance Enhancement (MSPE) program. MSPE is primarily based on the principles of Mindfulness-Based Stress Reduction (MBSR; Kabat-Zinn, 1990; Wong et al., 2022), whereas MAC is more closely aligned with Acceptance and Commitment Therapy (ACT; Hayes et al., 1999; Josefsson et al., 2020). The MAC model suggests that performance enhancement does not depend on continuous monitoring or suppression of negative experiences but rather on cultivating present-moment attention and emotional acceptance to achieve optimal functioning (Josefsson et al., 2019).

This theoretical stance stands in contrast to traditional Psychological Skills Training (PST) approaches, which have dominated sport psychology for several decades and are primarily grounded in cognitive-behavioral theory (Meichenbaum, 1973; McCarthy et al., 2023). PST emphasizes developing self-control over internal states—such as thoughts, emotions, and physiological responses—to improve athletic performance. Numerous studies have shown that PST can reduce negative internal states such as performance anxiety and enhance positive ones such as confidence (Lee et al., 2023). However, only a limited number of studies have demonstrated how these internal changes translate into actual performance improvements (see Gardner & Moore, 2006; Gardner, 2009), and many athletes report difficulty in consciously controlling their cognitive processes using traditional PST methods.

A large body of research has demonstrated that mindfulness training exerts positive effects across different populations (Kaufman et al., 2009; Keng et al., 2011; Oguntuase and Sun, 2022). In the field of sport psychology, Kabat-Zinn et al. were the first to introduce mindfulness meditation into rower training, and in recent years scholars have increasingly focused on mindfulness-based programs such as the MAC approach for their potential to enhance athletes’ performance and technical skills (Kaufman et al., 2018). Gooding et al. reported that dispositional mindfulness significantly predicted free-throw accuracy among collegiate basketball players, alongside practice frequency and years of competitive experience (Gooding and Gardner, 2009). From a mechanistic perspective, mindfulness training may activate specific brain regions that influence the use of emotion regulation strategies, thereby promoting both physical and psychological well-being. Evidence further suggests that individuals with higher levels of mindfulness are more likely to employ adaptive strategies such as cognitive reappraisal (Junça-Silva and Caetano, 2023), remain focused on the present without distraction from irrelevant thoughts, and engage in more objective analyses of ongoing events, which facilitates positive appraisal and integration of personal experiences (Lehman et al., 2025). Taken together, these findings indicate that mindfulness is not only closely related to athletic performance but also plays a significant predictive role in the use of emotion regulation strategies.

Although previous meta-analytic evidence has examined the role of mindfulness-based strategies in emotion regulation more broadly, the scope has remained relatively general. For example, Raugh and Strauss (2024) conducted a systematic review and meta-analysis synthesizing the implementation of mindfulness-based emotion regulation strategies across diverse populations. While their findings provide important insights into the effectiveness of mindfulness in enhancing regulatory capacities, the review encompassed heterogeneous samples and did not specifically target athletes. Moreover, their inclusion criteria were not restricted to randomized controlled trials (RCT) or well-designed intervention studies, which limits the strength of causal inferences. Existing reviews have either focused on general populations and addressed broader psychological outcomes such as stress, anxiety, or well-being, without treating emotion regulation as a central construct (Bühlmayer et al., 2017). Consequently, the unique implications of mindfulness interventions for emotion regulation within athletic contexts remain underexplored. The present study addresses this gap by conducting the first meta-analysis specifically focused on the effects of mindfulness interventions on emotion regulation in athletes. By restricting inclusion to intervention studies with pre–post control group designs and extractable effect sizes, the analysis provides evidence regarding the causal impact of mindfulness-based programs on athletes’ regulatory capacities. In addition, within the mainstream intervention approaches in sport psychology, PST has long been regarded as a core method for enhancing athletes’ psychological regulation. PST typically includes techniques such as self-talk, imagery, attentional control, and relaxation training. A randomized controlled trial by Röthlin et al. (2020) found that both the MAC intervention and a multimodal PST program (including arousal regulation, imagery, self-talk, and goal setting) produced beneficial effects on emotion regulation, attentional control, and acceptance of emotions compared with a waitlist control group. However, compared with both PST and control groups, participants receiving MBIs reported reduced experiential avoidance, and manipulation checks indicated greater improvements in mindfulness following MBI, whereas PST interventions facilitated greater use of psychological skills. These findings suggest that MBIs and PST may yield both distinct and overlapping positive outcomes. From a theoretical perspective, mindfulness-based and traditional psychological training approaches differ substantially in their foundational principles, mechanisms of action, and regulatory pathways. MBIs emphasize present-moment awareness and nonjudgmental acceptance, promoting self-regulation by enhancing emotional awareness and reducing reactivity. In contrast, PST focuses on goal-directed cognitive control, optimizing performance through strengthened attentional focus and self-instruction. However, to date, no integrative meta-analysis has directly compared the differential effects of these two intervention paradigms, and it remains unclear whether MBIs and PST exert distinct influences on athletes’ emotion regulation. Building upon existing meta-analytic evidence on mindfulness interventions, the present study extends the scope to a comparative meta-analysis, systematically examining the differential effects of MBIs and traditional PST on emotion regulation among athletes. Such a direct comparison not only clarifies the relative strengths and contextual applicability of both approaches but also provides evidence-based insights for the advancement of psychological training practices in sport.

## Methods

### Search strategy

The study was conducted in strict accordance with the PRISMA (Preferred Reporting Items for Systematic Reviews and Meta-Analyses) guidelines (Page et al., 2021). The review protocol was pre-registered on PROSPERO (CRD420261340782). The literature search for this study was conducted across multiple databases, including Web of Science, PubMed, PsycINFO, and Scopus, with a cutoff date of March 6, 2026. Database-specific search strings were constructed using Boolean operators to combine three conceptual blocks: (1) mindfulness intervention terms, (2) athlete/sport population terms, and (3) emotion regulation outcome terms.

Studies were selected based on the following criteria: First, the study must employ a pre-post controlled experimental design, including at least one intervention group and one control group, with data comparisons before and after the intervention. Second, the intervention method must be mindfulness-based. Third, the study must report pre- and post-intervention data for emotion regulation variables, clearly indicating the measurement tools used, with complete and extractable data to allow for the calculation or extraction of effect sizes. Fourth, the participants must be athletes, and athletes should be the primary focus of the study. Finally, only peer-reviewed journal articles were included, with conference papers, theses, and other non-peer-reviewed publications excluded from selection.

### Coding procedure

Two researchers independently assessed the methodological quality of the included studies. The methodological quality was assessed using the RoB 2 tool for randomized controlled trials (RCTs; Higgins et al., 2011) and the ROBINS-I tool for quasi-experimental studies. In case of any disagreement between the two primary researchers, a third researcher was consulted to reach a consensus.

In the literature coding process of this study, the basic information of each study was first recorded, including author details and publication year. The sample sizes for both the intervention and control groups in each independent sample were documented to ensure accurate comparison analysis. Effect sizes (such as standardized mean differences, SMD) and their corresponding standard errors were considered core data, and these were annotated alongside the intervention duration and intervention protocol. Crucially, the intervention protocols were categorized and coded to facilitate comparative analysis, specifically distinguishing between the Mindfulness-Acceptance-Commitment (MAC) approach, Mindful Sport Performance Enhancement (MSPE), and traditional Psychological Skills Training (PST). Effect sizes (e.g., standardized mean differences, SMD), standard errors, intervention duration, and the names of emotion regulation measurement tools (e.g., DERS, MAAS, ERQ) were also extracted and coded to support subsequent meta-analytic comparisons.

### Data analysis

This study’s meta-analysis was conducted using the meta and meta for packages in RStudio. These packages are widely used for conducting meta-analytic calculations, including effect size estimation, heterogeneity analysis, and model fitting. The meta package was primarily used for basic meta-analysis procedures such as effect size calculation, while the meta for package was employed for more advanced statistical modeling, including random-effects and fixed-effects models, as well as the assessment of publication bias and heterogeneity across studies.

## Results

### Study selection

**Figure 1:**
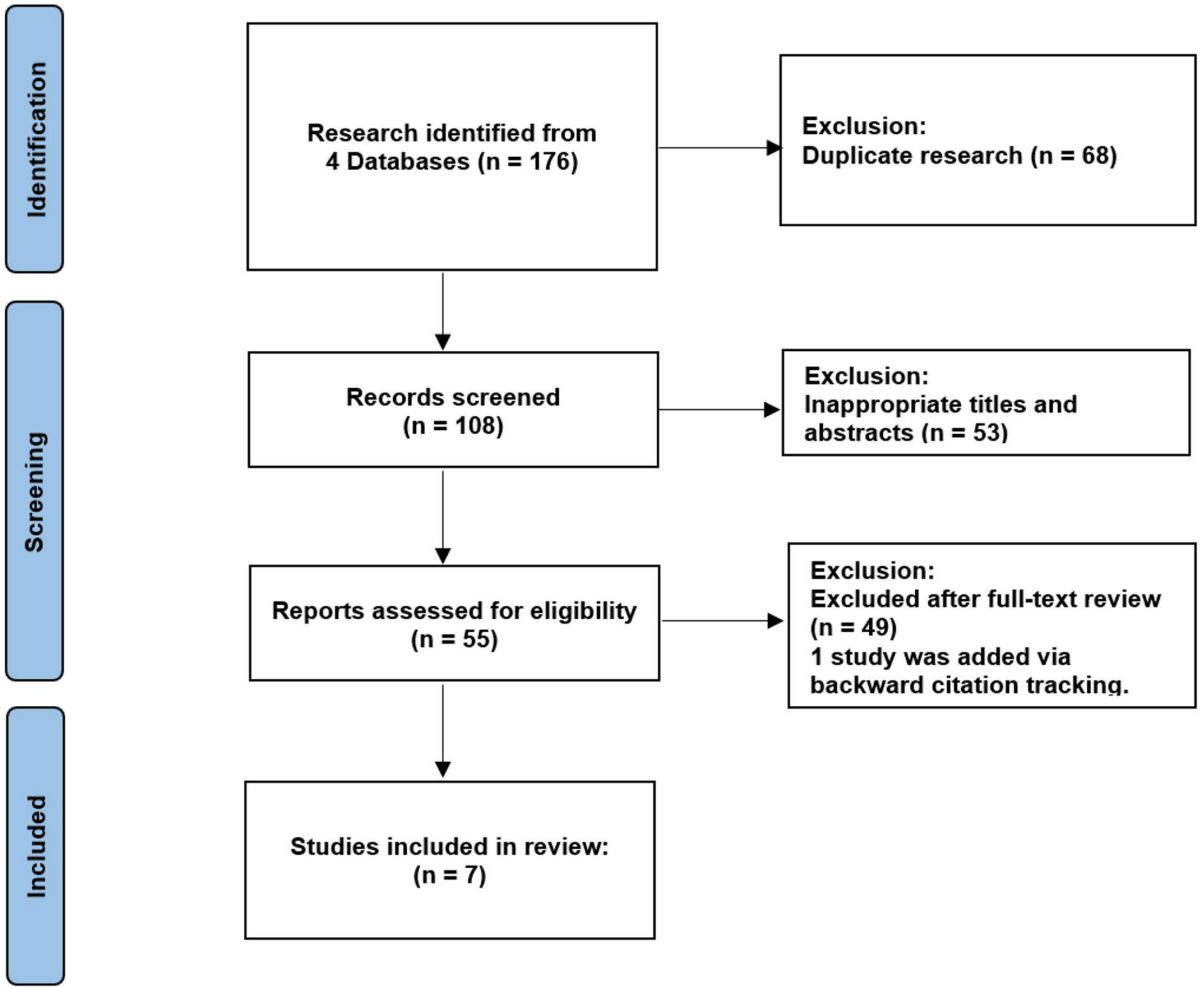
PRISMA 2020 flow diagram.

The study was conducted in strict accordance with the PRISMA guidelines (Page et al., 2021). The final analysis included 7 studies that met the criteria, with a total of 7 effect sizes and 273 participants. A total of 176 relevant articles were initially identified. After removing 68 duplicates using Zotero, 53 articles were excluded by screening titles and abstracts. The full texts of the remaining 55 articles were reviewed, and 6 studies met the predefined inclusion and exclusion criteria. Additionally, 1 study was identified through backward citation tracking from relevant systematic reviews and meta-analyses. In total, 7 articles were included in the final analysis, all of which were published in peer-reviewed English journals (Figure 1). Among them, three studies used traditional psychological skills training interventions as control conditions, allowing for a comparative examination of the differential effects between mindfulness-based interventions and traditional psychological training on athletes’ emotion regulation outcomes.

### Quality assessment and basic characteristics of included studies

After evaluation by two coders, it was found that most of the included studies had certain limitations in the randomization process or blinding implementation. As a result, among the five RCTs, Josefsson et al. (2019) was identified as having a “low risk” of bias, while three studies (Samadi et al., 2020; Emami et al., 2025; Gross et al., 2018) were rated as having “some concerns.” Hut et al. (2023) was categorized as “high risk.” Regarding the two quasi-experimental studies, Minkler et al. (2024) exhibited a “moderate risk” of bias, and Kelemen et al. (2025) was judged to have “some concerns” according to ROBINS-I criteria.

**Figure 2:**
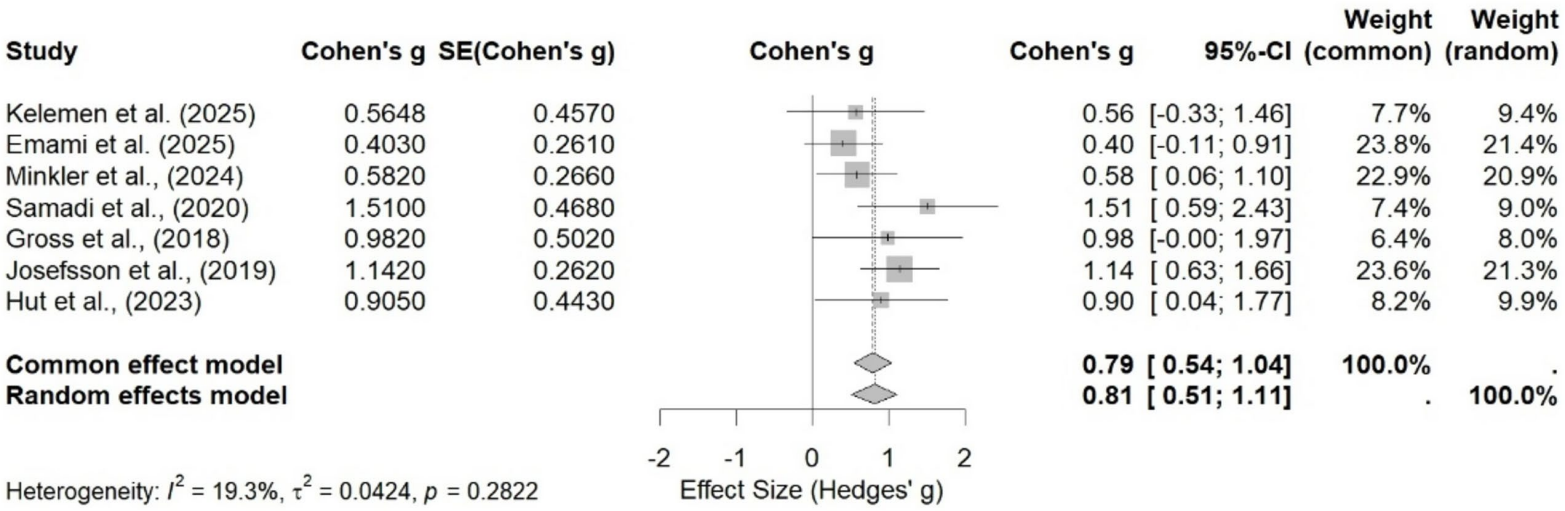
Forest plot of the overall effects of mindfulness-based interventions.

**Figure 3:**
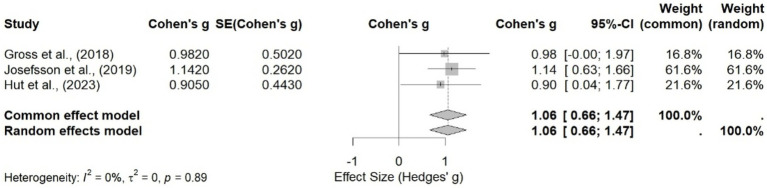
Forest plot of MAC VS PST intervention.

Across the included studies, participants comprised a heterogeneous range of athletic populations, including football players, basketball players, endurance runners, track and field athletes, and mixed-sport athlete samples, with competitive levels ranging from beginner and collegiate athletes to elite athletes. The majority of studies employed randomized controlled trial (RCT) designs (five studies), while two adopted quasi-experimental designs. Regarding interventions, two studies implemented the Mindful Sport Performance Enhancement (MSPE) program, three utilized Mindfulness-Acceptance-Commitment (MAC) training, one applied a general mindfulness-based intervention (MBI), and one combined mindfulness training with sleep hygiene education (MPSH). Intervention durations varied from 6 to 10 weeks, with most studies adopting protocols between 6 and 8 weeks. In terms of outcome measures, the Difficulties in Emotion Regulation Scale (DERS) and its short form (DERS-SF) were most frequently used, while the Emotion Regulation Questionnaire (ERQ) was used in one study. Collectively, these studies assessed both general and sport-related aspects of emotion regulation, providing a heterogeneous but complementary set of measurement approaches. To provide a directly inspectable overview of this heterogeneity, Table 1 summarizes the sport type, competitive level, sample size, study design, intervention protocol, control condition, intervention dose, outcome measure, assessment time point, randomization, assessor blinding, risk of bias, and attrition rate for each included study.

**Table 1:** Characteristics of included studies, athlete populations, intervention protocols, and methodological features.

Author	N	Population	Level	Design	Intervention	Control condition	Dose	Measures	Outcome time point	Randomization	Assessor blinding	Risk Of bias	Attrition rate
Kelemen et al. (2025)	20	endurance runners	Elite	Quasi-experimental	MSPE	Wellness Program	6 wks × 1/wk. × 70–90 min	DERS	Post-test + 3-month follow-up	Yes	NR	Some concerns	NR
Minkler et al. (2024)	59	Mixed athletes	Collegiate	Quasi-experimental	MSPE	Waitlist control	6 wks × 1/wk. × 60 min	DERS	Post-test	No	Self-report	Moderate risk	9.2%
Samadi et al. (2020)	24	Endurance runners	Beginner	RCT	MBI	No-treatment control	6 wks × 1/wk. × 70–90 min	ERQ	Post-test	Yes	Self-report	Some concerns	0%
Emami et al. (2025)	60	Football players	Elite	RCT	MPSH	Wellness Program	8 wks × 1/wk. × 90 min	DERS	Post-test	Yes	Self-report	Some Concerns	0%
Gross et al. (2018)	18	Basketball players	Collegiate	RCT	MAC	PST	6 wks × 1/wk. × 60 min	DERS	Post-test	Yes	Self-report	Some Concerns	9.1%
Josefsson et al. (2019)	68	Mixed athletes	Elite	RCT	MAC	PST	7 wks × 1/wk. × 50 min	DERS	Post-test	Yes	Self-report	Low risk	19.1%
Hut et al. (2023)	24	Track and field athletes	Collegiate	RCT	MAC	PST	6 wks × 1/week × 60 min	DERS-SF	Post-test	Yes	Self-report	High risk	20%

MSPE, mindful sport performance enhancement; MPSH, mindfulness plus sleep hygiene education; MBI, mindfulness-based intervention; MAC, mindfulness–acceptance–commitment; DERS, difficulties in emotion regulation scale; DERS-SF, difficulties in emotion regulation scale–short form.

### Overall effects

A meta-analysis was conducted to synthesize the effect sizes from all eligible mindfulness-based intervention protocols, including MSPE, MAC, and general MBI programs. This overall analysis comprised 7 studies (*k* = 7) and employed the Restricted Maximum Likelihood (REML) method to estimate the variance between studies. The summary results revealed a statistically significant overall effect (Hedges’ *g* = 0.81, 95% CI [0.51, 1.11], *p* < 0.0001), initially suggesting that mindfulness-based interventions may have a moderate positive effect on emotion regulation in athletes compared with control conditions (Figure 2).

In contrast, the MAC versus PST comparison was conducted only in the subset of studies that directly compared Mindfulness–Acceptance–Commitment (MAC) training with traditional Psychological Skills Training (PST). In this small subset of comparative studies, the pooled Hedges’ *g* was 1.06 (95% CI [0.66, 1.47], *z* = 5.17, *p* < 0.001), initially suggesting a larger effect estimate for MAC than for PST. Because this comparison was based on only three studies, the result should be interpreted as exploratory rather than conclusive (Figure 3).

### Heterogeneity analysis

The heterogeneity between effect sizes across the included studies was assessed. Cochran’s *Q* test yielded a non-significant result (Q (6) = 7.44, *p* = 0.28), suggesting that there was no significant heterogeneity between the studies. Further heterogeneity indices supported this conclusion: the estimated total heterogeneity was negligible (*τ*^2^ = 0.04, *I*^2^ = 19.3%, *H*^2^ = 1.11), suggesting that the observed between-study variability was limited, although this finding should be interpreted cautiously given the small number of included studies.

The heterogeneity of effect sizes across the studies comparing Mindfulness–Acceptance–Commitment (MAC) and Psychological Skills Training (PST) on athletes’ emotion regulation was examined. There was no significant heterogeneity among the included studies [Q (2) = 0.24, *p* = 0.89; *τ*^2^ = 0.00; *I*^2^ = 0.0%; *H*^2^ = 1.00].

Overall, these findings highlight that MAC provides enhancement of emotion regulation in athletes than traditional PST programs.

### Publication bias

**Figure 4:**
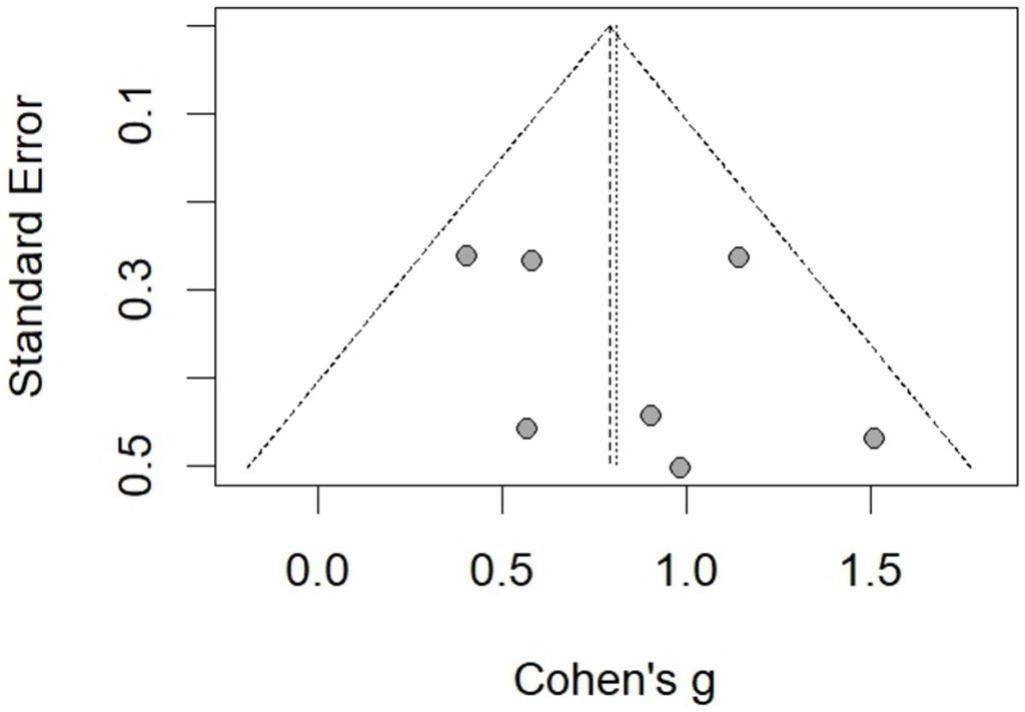
Funnel plot of the overall mindfulness-based intervention analysis.

**Figure 5:**
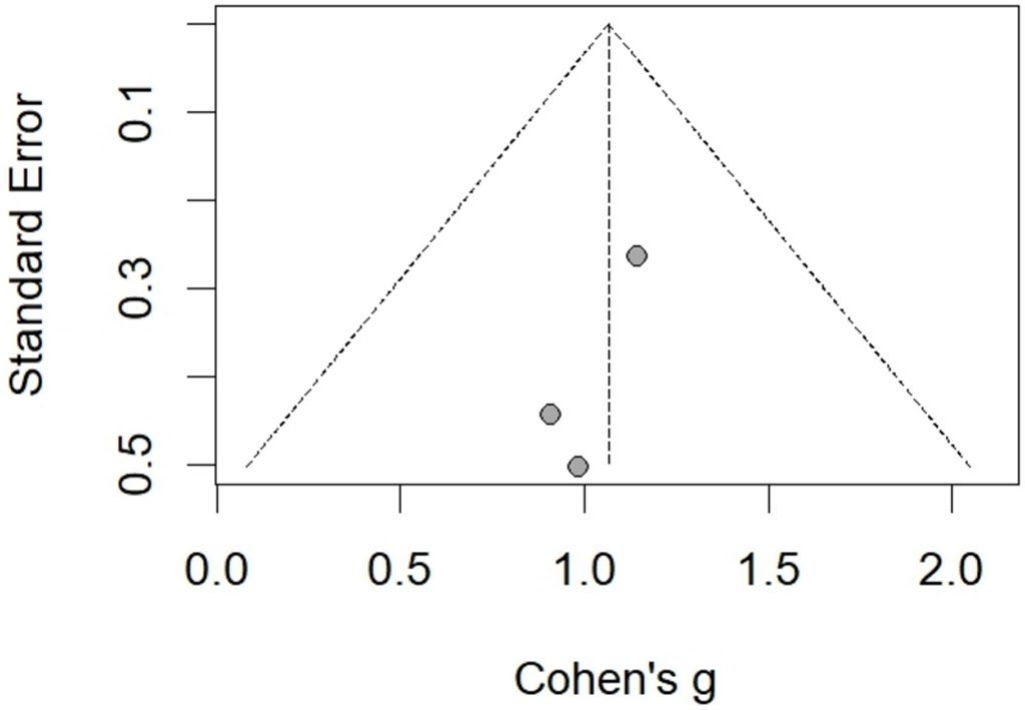
Funnel plot of MAC VS PST intervention.

**Figure 6:**
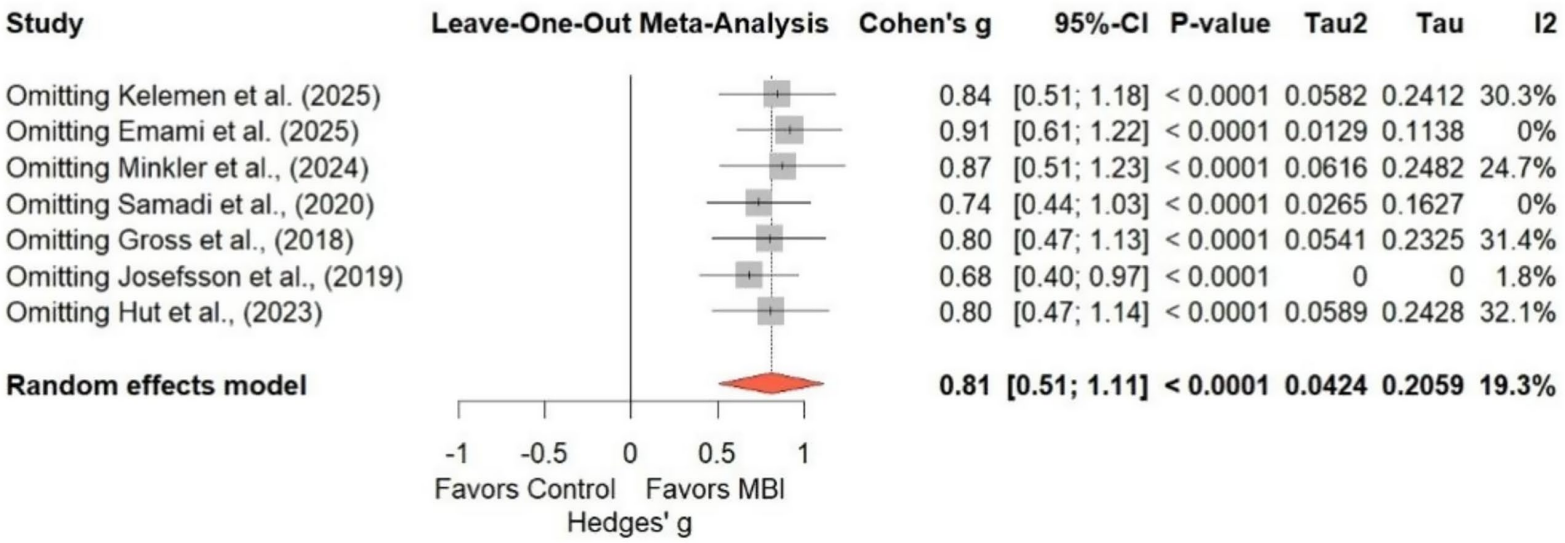
Leave-one-out sensitivity analysis.

Based on the study results, we plotted a funnel plot (Figures 4, 5). However, using a funnel plot to assess publication bias can be subjective and does not provide a precise test of publication bias. Therefore, to conduct an objective evaluation, we performed Egger’s regression test to assess whether publication bias was present in the meta-analysis. The results indicated a regression coefficient of *z* = 0.88, with *p* = 0.38. No statistically significant evidence of publication bias was detected; however, given the small number of included studies, these results should be interpreted cautiously and cannot rule out potential small-study effects or selective reporting. Similarly, in the meta-analysis comparing Mindfulness–Acceptance–Commitment (MAC) and Psychological Skills Training (PST) on athletes’ emotion regulation, Egger’s regression test also revealed no evidence of publication bias (*z* = −0.45, *p* = 0.65). However, because the number of included studies was small, especially in the comparative analysis, the non-significant Egger’s test should be interpreted cautiously and cannot rule out selective reporting or small-study effects (see Figure 6).

### Subgroup and moderator analyses

Although the initial protocol considered exploring potential moderating factors, no formal subgroup analyses were performed in the present meta-analysis. This decision was primarily informed by the limited number of included studies (*k* = 7). Given this small sample size, partitioning the data by sport type, intervention duration, or athlete level would result in insufficient statistical power, potentially leading to biased or misleading interpretations. While no significant heterogeneity was detected (*τ*^2^ = 0.04, *I*^2^ = 19.3%, *H*^2^ = 1.11), the current findings are presented as a preliminary overall estimate rather than as evidence of a robust effect across moderator-defined subgroups.

### Sensitivity analysis

To assess the robustness of the overall effect size estimate, a leave-one-out sensitivity analysis was conducted in which each study was systematically removed from the analysis and the remaining studies were re-pooled. The pooled effect size remained statistically significant across all iterations (all *p* < 0.0001), ranging from Hedges’ *g* = 0.68 (95% CI [0.40, 0.98]) to Hedges’ *g* = 0.92 (95% CI [0.61, 1.22]), closely approximating the overall estimate of Hedges’ *g* = 0.809 (95% CI [0.51, 1.11]). No single study was identified as an outlier or exerted undue influence on the overall conclusion, supporting the stability and robustness of the findings.

## Discussion

This study demonstrates that mindfulness-based interventions (MBIs) exert a significant positive impact on athletes’ emotional regulation. Overall, MBIs showed a moderate effect, which is consistent with prior evidence on the beneficial effects of mindfulness interventions in athletic populations (Bühlmayer et al., 2017). At the same time, the findings converge with research from broader domains, where MBIs have been shown to enhance individuals’ capacity for emotional regulation and support more effective management of stress and anxiety (Raugh et al., 2024).

Although statistical heterogeneity was low and non-significant across analyses (overall: *I*^2^ = 19.3%, *τ*^2^ = 0.04, Q (6) = 7.44, *p* = 0.28; MAC vs. PST subgroup: *I*^2^ = 0.0%, *τ*^2^ = 0.00, Q (2) = 0.24, *p* = 0.89), these estimates should be interpreted with caution for several methodological reasons. The Q test has well-documented limitations in statistical power when the number of included studies is small (Higgins and Thompson, 2002; Ioannidis et al., 2007). With only seven effect sizes in the primary analysis and three in the MAC versus PST subgroup, the Q test is substantially underpowered to detect true between-study variance. A non-significant result therefore cannot be taken as evidence of genuine homogeneity. Regarding publication bias, Egger’s test did not reach statistical significance, and the funnel plot did not reveal obvious visual asymmetry, providing no strong evidence of small-study effects. However, Egger’s test has been shown to have low statistical power when fewer than ten studies are included (Sterne et al., 2011), and a non-significant result under these conditions provides limited reassurance against publication bias. The funnel plot should therefore be regarded as exploratory. Additionally, all included studies reported significant or positive findings, and no null-result studies were identified, which may reflect selective publication of favorable outcomes. Beyond statistical heterogeneity, several important sources of clinical heterogeneity warrant consideration. First, the included studies employed four distinct intervention protocols—MSPE, MAC, MBI, and MPSH—which differ substantially in theoretical foundations, session content, and mechanisms of change. Notably, the MPSH protocol (Emami et al., 2025) incorporated sleep hygiene education as an additional active component, making it difficult to attribute observed effects solely to mindfulness training. Second, intervention duration ranged from 6 to 10 weeks, with session lengths varying from 50 to 90 min. Third, participant competitive levels spanned from recreational to elite professional athletes, encompassing individual and team sports across multiple cultural contexts. Fourth, emotion regulation was assessed using instruments with different conceptual foundations: three studies used the full 36-item DERS, two used the DERS-SF, one used a modified 4-subscale DERS (Josefsson et al., 2019), and one used the ERQ (Samadi et al., 2020). The ERQ measures cognitive reappraisal and expressive suppression strategy use, whereas the DERS family assesses regulation difficulties—these instruments are not directly comparable. Taken together, the pooled effect size should be interpreted as a broad estimate across a diverse set of interventions and populations. Although subgroup analyses were planned, the limited number of included studies (*k* = 7) rendered such analyses insufficiently powered to yield reliable estimates. From a practical perspective, these results underscore the utility of incorporating mindfulness training into athletes’ psychological preparation and mental skills programs. Coaches, sport psychologists, and practitioners can consider structured mindfulness-based protocols, such as MAC or MBSR, as effective tools to enhance emotional regulation, resilience, and performance stability. The moderate effect size further suggests that MBIs may serve as a complement to existing psychological skills training, offering a non-invasive, low-cost, and adaptable approach suitable across a range of sports.

The exploratory comparative analysis initially suggested a larger effect estimate for mindfulness-based interventions than for PST in athletes’ emotion regulation. However, because this comparison was based on a very small subset of studies, it should not be interpreted as conclusive evidence that mindfulness-based interventions are more effective than PST. Theoretically, PST primarily relies on volitional control and cognitive reappraisal strategies, aiming to maintain an optimal psychological state by actively regulating or replacing undesirable emotions. In contrast, the MAC approach emphasizes awareness and acceptance, helping individuals change their relationship with emotions rather than the emotional content itself, thereby reducing internal struggle and suppression. As Birrer et al. (2012) pointed out, excessive pursuit of psychological control can paradoxically impair performance because such control consumes limited attentional resources and increases self-focused attention, which in turn interferes with the smooth execution of automatized skills. Conversely, the MAC approach fosters metacognitive awareness and psychological flexibility, enabling athletes to remain aware rather than resistant when experiencing negative emotions, allowing emotions to naturally arise and pass. This awareness–acceptance mechanism is particularly critical in high-pressure, dynamic, and unpredictable competitive environments. Compared with the “control-oriented” strategies of PST, MAC aligns more closely with the psychological principles of automatic skill execution and cognitive resource efficiency, reducing cognitive overload, sustaining task-focused attention, and enhancing situational adaptability. Theoretically, mindfulness-based interventions may help athletes maintain automatic performance and high-level functioning in complex and changing environments by reducing rumination, decreasing experiential avoidance, and optimizing cognitive resource allocation (Josefsson et al., 2019).

Publication bias was assessed using a funnel plot and Egger’s test for funnel plot asymmetry. The funnel plot did not reveal obvious visual asymmetry, and Egger’s test did not reach statistical significance, suggesting no strong evidence of small-study effects in the current dataset. However, these findings must be interpreted with considerable caution. Egger’s test has been shown to have low statistical power when fewer than ten studies are included in a meta-analysis (Sterne et al., 2011), which is the case in the present review. Under these conditions, a non-significant Egger’s test result provides limited reassurance against publication bias, as the test is unlikely to detect asymmetry even if it is present. The funnel plot should therefore be regarded as exploratory rather than confirmatory, and the possibility of publication bias cannot be ruled out. Consistent with this concern, all included studies reported significant or positive findings for at least one primary outcome, and no studies with null or negative results for mindfulness interventions on athlete emotion regulation were identified in the search, which may reflect selective publication of favorable outcomes. The small number of included studies limits the strength of the evidence and reduces the generalizability of the present findings. In addition, publication bias tests such as Egger’s regression have limited statistical power when fewer than ten studies are included; therefore, the non-significant results should not be interpreted as definitive evidence of absence of publication bias.

It is noteworthy that only three randomized controlled trials (RCTs) to date have directly compared mindfulness-based interventions with traditional PST among athlete populations (Gross et al., 2018; Josefsson et al., 2019; Hut et al., 2023). These studies generally suggested larger improvements in mindfulness-related and emotion regulation outcomes in the MAC groups than in the PST groups; however, due to small sample sizes, variability in intervention formats, and differences in outcome measures, the current evidence base remains preliminary. Future research should employ larger, more diverse athlete samples, include cross-cultural comparisons, and adopt longitudinal RCT designs to further strengthen the evidence base for MAC and elucidate its long-term mechanisms in enhancing emotion regulation and athletic performance. Despite these promising findings, several limitations warrant consideration. Although the present meta-analysis provides preliminary evidence for the effectiveness of mindfulness-based interventions on athletes’ emotion regulation, several limitations should be noted. First, the number of eligible studies was relatively small, with a total sample size of only 273 athletes. The limited body of evidence constrains the generalizability of the findings and reduces the ability to examine potential moderators, such as sport type, competitive level, or intervention duration. Second, the outcome measures were primarily self-report questionnaires such as the DERS or ERQ. While these tools provide valuable insights into subjective experiences, they may be influenced by social desirability or self-perception biases and do not capture physiological or behavioral dimensions of emotion regulation.

It should be emphasized that the present meta-analysis focused on emotion regulation outcomes rather than direct measures of athletic performance. Therefore, the observed effects should not be interpreted as evidence that mindfulness interventions directly improve sport performance. One possible pathway is that mindfulness training may support more adaptive emotion regulation under competitive pressure by enhancing interoceptive awareness, attentional control, and acceptance of internal states. Consistent with this interpretation, Campo et al. (2026) reported that mindfulness-based training in athletes significantly improved interoceptive awareness, suggesting a potential mind–body mechanism through which mindfulness may influence emotion regulation and performance-related processes. However, whether these changes translate into measurable improvements in athletic performance remains an important question for future research. Future studies should address this issue by incorporating objective performance-based outcomes, such as race times or sport-specific performance indicators, alongside self-report measures of emotion regulation. In addition, physiological indices such as heart rate variability, cortisol levels, or EEG markers may help clarify the mechanisms through which mindfulness influences emotion regulation and performance-related processes. Larger randomized controlled trials with active control groups, appropriate follow-up assessments, and careful control of potential confounding variables, such as training volume, injury status, and competition exposure, are needed to determine not only whether mindfulness interventions are effective, but also how and for whom they may be most beneficial.

## Conclusion

Overall, this meta-analysis provides preliminary evidence that mindfulness-based interventions may enhance emotion regulation among athletes. Given the limited number of included studies, the present findings should be verified through larger-sample, higher-quality randomized controlled trials. The results initially suggest a significant, moderate overall effect of mindfulness on emotion regulation, based on seven studies. Mindfulness-based approaches may be associated with larger improvements than traditional PST in promoting adaptive emotion regulation; however, this comparison was based on only three studies and should be interpreted with caution. These findings need further examination before firm conclusions can be drawn about the comparative advantage of mindfulness-based interventions. Although no significant heterogeneity or publication bias was detected, these results should be interpreted cautiously given the limited number of included studies and the low statistical power of the corresponding tests. Future research should further examine potential moderating factors across different athlete populations and intervention designs, clarify the boundary conditions of the construct by employing conceptually consistent outcome measures, and explore the long-term effects and mechanisms of mindfulness-based interventions in competitive sport contexts.

## Data Availability

The original contributions presented in the study are included in the article/supplementary material, further inquiries can be directed to the corresponding author.
